# Aerophobin-1 from the Marine Sponge *Aplysina* *aerophoba* Modulates Osteogenesis in Zebrafish Larvae

**DOI:** 10.3390/md20020135

**Published:** 2022-02-11

**Authors:** Marta Carnovali, Maria Letizia Ciavatta, Ernesto Mollo, Vassilios Roussis, Giuseppe Banfi, Marianna Carbone, Massimo Mariotti

**Affiliations:** 1 IRCCS Istituto Ortopedico Galeazzi, Via R. Galeazzi 4, 20161 Milano, Italy; marta.carnovali@grupposandonato.it (M.C.); banfi.giuseppe@unisr.it (G.B.); 2 Consiglio Nazionale delle Ricerche—Istituto di Chimica Biomolecolare (CNR-ICB), Via Campi Flegrei 34, 80078 Pozzuoli (NA), Italy; lciavatta@icb.cnr.it (M.L.C.); emollo@icb.cnr.it (E.M.); 3 Section of Pharmacognosy and Chemistry of Natural Products, Department of Pharmacy, National and Kapodistrian University of Athens, Panepistimiopolis Zografou, 15771 Athens, Greece; roussis@pharm.uoa.gr; 4 School of Medicine, Vita-Salute San Raffaele University, Via Olgettina 58, 20132 Milano, Italy; 5 Dipartimento di Scienze Biomediche, Chirurgiche ed Odontoiatriche, Università Degli Studi di Milano, Via della Commenda 10, 20122 Milano, Italy

**Keywords:** marine natural products, bromotyrosine, osteogenesis, zebrafish, drug discovery

## Abstract

Longer life expectancy has led to an increase in efforts directed to the discovery of new healing agents for disorders related to aging, such as bone diseases. Harboring an incredible variety of bioactive metabolites, marine organisms are standing out as fruitful sources also in this therapeutic field. On the other hand, the in vivo zebrafish model has proven to be an excellent low-cost screening platform for the fast identification of molecules able to regulate bone development. By using zebrafish larvae as a mineralization model, we have thus evaluated the effects of the crude acetonic extract from the marine sponge *Aplysina aerophoba* and its bromotyrosine components on bone development. Obtained results led to the selection of aerophobin-1 (**1**) as a promising candidate for applications in regenerative medicine, paving the way for the development of a novel therapeutic option in osteoporosis treatment.

## 1. Introduction

The high structural diversity and bioactivity possessed by marine natural compounds have been proven straight off since the early studies leading to a relevant high number of marine metabolites that have been entered in the pharmaceutical industry pipeline [1,2,3]. Although treatment of infections and cancer represent the target of most of the studies aimed at exploring the pharmacological properties of compounds from the sea [4], the number of scientific papers dealing with marine natural products having modulatory effects on skeletal development has significantly increased in the recent years [5,6,7,8,9,10]. This tendency is also a consequence of the higher interest toward the discovery of new healing agents for disorders related to aging. In spite of this, however, the potential of marine chemical diversity to deliver pro-osteogenic agents still remains superficially explored [11].

On the other hand, with the aim at ensuring the widest exploitation of pharmacological potential held in marine bioresources, efforts are continuously directed toward the development of novel strategies for the rapid detection of promising bioactive natural products that can act as lead molecules for the development of new drugs [12]. In the classical phenotypic approaches for drug discovery, the zebrafish (*Danio rerio*) in vivo model offers a valid, fast, and low-cost screening platform for the identification of compounds mediating specific biological processes [13]. In particular, the rapid external development of the bone system along with the transparency of the body make zebrafish larvae especially suitable in the study of organogenesis and, in particular, osteogenesis [14]. In addition, the bone tissue of adult zebrafish is very similar to the human one for the presence of a mineralized matrix and functional osteoblasts and osteoclasts [15], while the process of embryonic osteogenesis is characterized by two different types of ossification: endochondral, which takes place from a cartilaginous scaffold, and intramembranous, which is driven by mesenchymal stem cell precursors [16,17]. All these features, along with the high genetic similarity between humans and zebrafish, with 70% of zebrafish genes having an identifiable human ortholog [18], make zebrafish an ideal model for studies in osteogenesis.

Capitalizing on this model, our ongoing marine bioprospecting programs are aimed at exploring in depth the potential of marine natural products as regulators of osteogenesis, including the screening of the chemical content of marine benthic invertebrates for their modulatory activity on the development of the zebrafish skeletal system. Herein we report the study of the osteogenic properties of both the crude acetonic extract from the Mediterranean sponge *Aplysina aerophoba* (Nardo, 1833) and its main bromotyrosine components with a special focus on intramembranous ossification.

## 2. Results

### 2.1. Toxicity and Osteogenic Activity of the Sponge Extract

**Figure 1:**
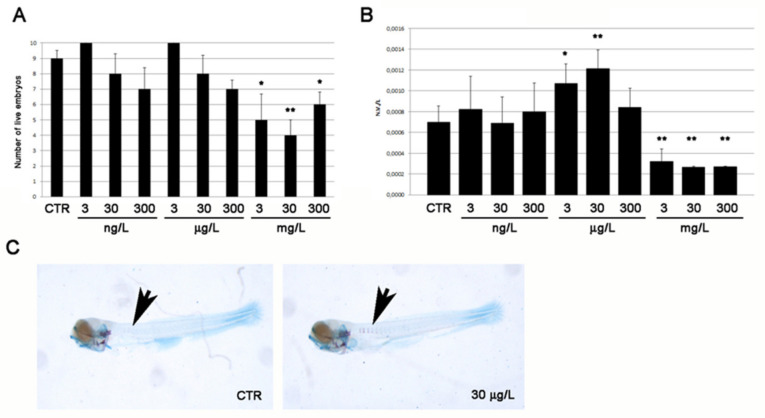
Effect of different dilutions of *A. aerophoba* crude extract on (**A**) zebrafish embryos/larvae viability and (**B**) mineralization rate of the vertebral bodies. (**C**) Mineralized vertebrae visualized by alizarine red staining (purple) together with unmodified cartilage structures (blue) in control (0 concentration) and treated (30 µg/L sponge extract) larvae. Significant differences were evaluated by using the Student’s *t*-test (α = 0.05; * *p* ≤ 0.05, ** *p* ≤ 0.01).

To assess the toxicity of the crude acetone extract of *A.* *aerophoba*, zebrafish embryos were exposed from 1 h post fertilization (hpf) up to 5 days post fertilization (dpf) to the sponge extract at 1:10 serial dilutions, starting from 0 (control = vehicle DMSO only) to 300 mg/L. A significantly increased mortality compared to control was observed at concentrations ≥ 3 mg/L (Figure 1A). In order to investigate the effects of the sponge extract on bone development, the intramembranous ossification in the entire larval body was analyzed, focusing on vertebral mineralization. The ratio between the number of vertebrae (NV) and total body length (L) was used as an index of vertebral mineralization rate. A significant increment of NV/L compared to control was measured at 3 and 30 μg/L concentrations of *A. aerophoba* extract (Figure 1B), as visualized by alizarin red live staining (Figure 1C).

### 2.2. Modulatory Effects of Pure Compounds ***1***–***8*** on Zebrafish Embryo Osteogenesis

**Figure 2:**
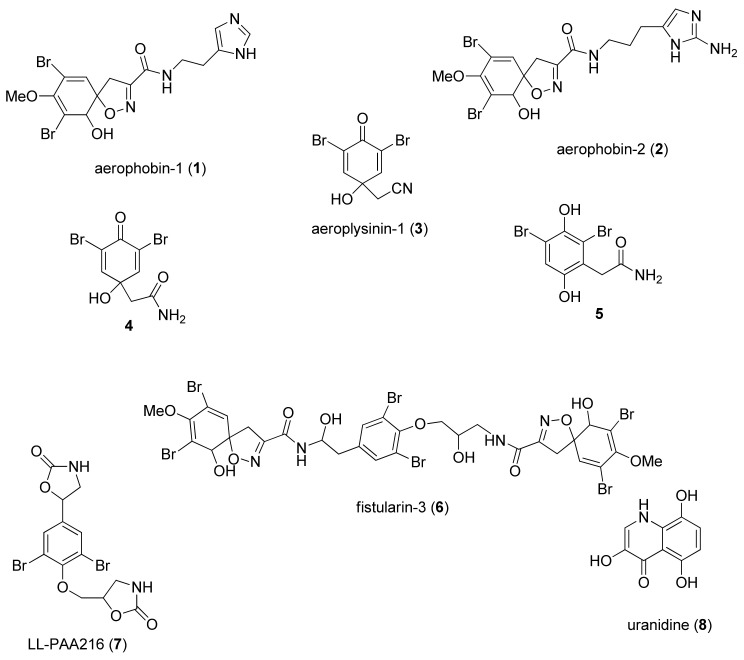
Chemical structures of bromotyrosine alkaloids isolated from *A. aerophoba*.

**Figure 3:**
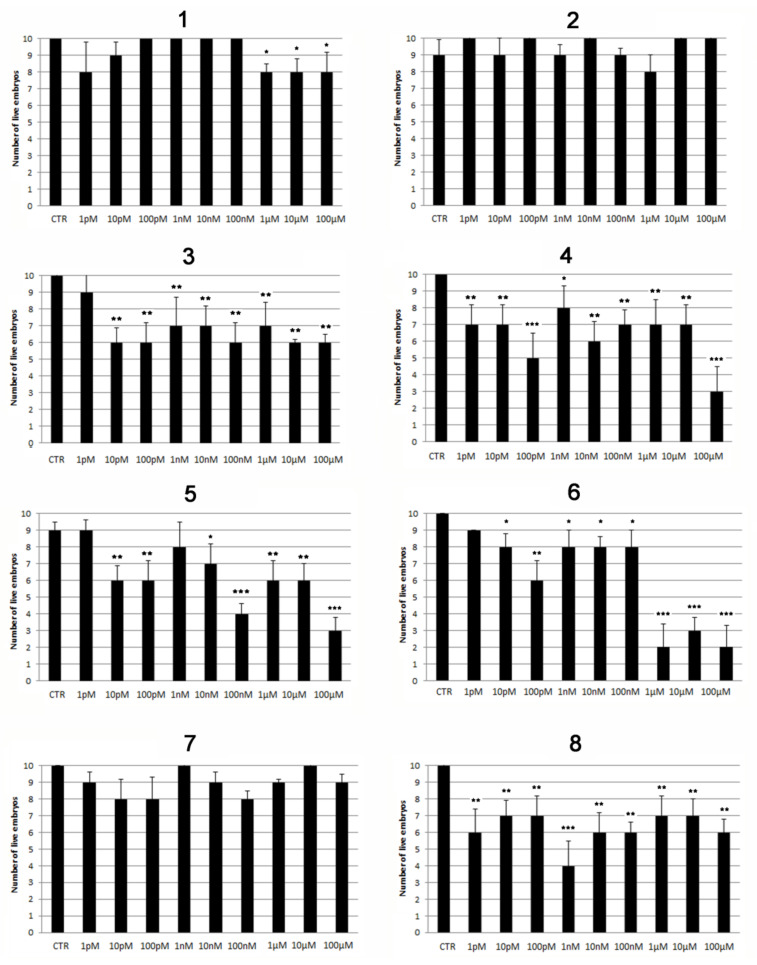
Viability of embryos treated with different concentrations (from 1 pM to 100 µM) of the eight compounds *(***1–8***)* isolated from *A. aerophoba*. Bars indicate the number of live embryos at 5 dpf. Significant differences were evaluated by using the Student’s *t*-test (α = 0.05; * *p* ≤ 0.05, ** *p* ≤ 0.01, *** *p* ≤ 0.001).

Fractionation of the acetone extract of *A. aerophoba* was carried out by means of different chromatographic techniques, resulting in the isolation and purification of a series of bromotyrosine alkaloids, which were identified by spectroscopic methods according to data previously reported in the literature (Figure 2, Supplementary Materials S1–S16 and Materials and Methods section). Compounds **1**–**7**, which were the main components of the bromotyrosine mixture, and the co-occurring yellow pigment uranidine (**8**), were assayed on zebrafish embryos/larvae to evaluate both their toxicity and pro-osteogenic properties. The viability of embryos/larvae treated with purified compounds **1**–**8** was evaluated during the initial developmental stage (from 1 hpf to 5 dpf) by exposing groups of 10 embryos to 1:10 serial dilutions from 1 pM to 100 µM of each compound. Slight toxic effects in the later development stage (5 dpf) were exerted by compounds **3**–**5** at almost all concentrations (Figure 3, Table 1), while aerophobin-1 (**1**) showed very low toxicity when assayed at concentrations over 1 µM. Null toxic effects were induced by aerophobin-2 (**2**) and LL-PAA216 (**7**) (Figure 3 and Table 1). Conversely, a significant toxicity profile was observed for uranidine (**8**) and fistularin-3 (**6**), both inducing early death. In particular, embryos died at all tested concentrations of compound **8**, whereas treatment with fistularin-3 (**6**) induced death at 48 hpf. The latter was found to cause evident pericardial edema (PE), particularly at the highest concentrations tested (Figure 3 and Table 1).

**Figure 4:**
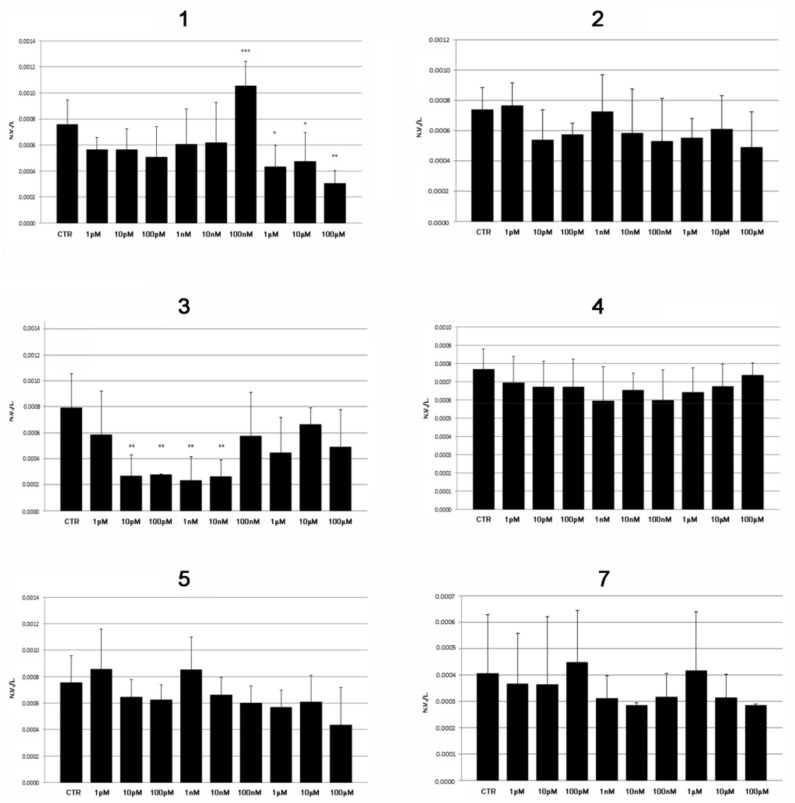
Mineralization rate in embryos exposed to compounds **1**–**5** and **7**, evaluated as number of mineralized vertebrae at 5 dpf normalized for embryo length (NV/L). Significant differences were evaluated by using the Student’s *t*-test (α = 0.05; * *p* ≤ 0.05, ** *p* ≤ 0.01, *** *p* ≤ 0.001).

Based on preliminary toxicological evaluations, the study of the osteogenic properties was limited to compounds **1**–**5** and **7**. The number of mineralized vertebrae (NV) was recorded in embryos treated with 1:10 dilutions from 1 pM to 100 µM of pure compounds **1**–**5** and **7** as well as in untreated embryos (control group = vehicle DMSO only). The recorded data were normalized with the total length of the larval body (L). A significant increase in the vertebral mineralization ratio (NV/L) was detected only in the embryos exposed to aerophobin-1 (**1**), with higher significance found at the minimum effective dose of 100 nM (Figure 4). On the other hand, as expected, an inhibitory effect on osteogenesis was reported for both aerophobin-1 (**1**) and (+)-aeroplysinin (**3**), in the range of their toxic concentrations (Figure 4).

## 3. Discussion

Besides having contributed to elucidating the mechanisms of formation of the vertebrate skeleton [19], studies on marine sponges have highlighted the ability of these organisms or of their associated microbes to produce bioactive molecules able to interfere with bone metabolism [6,7,8]. Most of this research has been carried out in vitro or by using mice as in vivo models. Instead, in our search for pro-osteogenic agents in marine sponges, we have taken advantage of the zebrafish embryo model. In preliminary screening, the crude acetone extract of *A. aerophoba* induced pro-osteogenic effects on zebrafish larvae bone development. This prompted us to investigate the chemical content of the sponge, with the aim of identifying the active components responsible for the observed effects. The study resulted in the isolation of a pool of known bromotyrosine alkaloids (**1**–**7**), according to the literature for the genus *Aplysina* [20]. This class of metabolites constitutes a geographically widespread class of compounds unique to the phylum Porifera, which has attracted the interest of both chemists and pharmacologists for their high structural diversity and bioactivity. Such a structural variety ranges from simple monomeric structures as the isolated compounds **3**–**5** to dimeric or more complex architectures, such as those displayed by suberedamine [21] and fistularin-3 (**6**), respectively. It has been postulated that these compounds are produced after sponge tissue injury as chemical defense and/or are released in seawater to protect *Aplysina* sponges from the invasion of microorganisms or animal predators [22,23]. Besides their ecological role, this class of marine alkaloids is characterized by a wide spectrum of pharmacological activities, including antitumor, anti-inflammatory, and neurological properties, as well as antimicrobial actions [24,25,26]. On the other hand, their possible effects on bone development have never been reported to date. In the present report, the bromotyrosine alkaloids **1**–**7** along with the yellow pigment uranidine (**8**) [27] have been firstly assayed for their toxicity on zebrafish embryos. Only aerophobins-1 (**1**) and -2 (**2**) and LL-PAA216 (**7**) displayed low toxicity, whereas compounds **4**–**6** and **8** were toxic in a wide spectrum of concentration, with uranidine (**8**) having the worst toxicity profile. It is worth noting, however, that in a recent study, fistularin-3 (**6**) and LL-PAA216 (**7**) were not toxic on adult zebrafish at 0.1 mg/mL (100μM), though exerting neurological activity resulting in a sedative effect [28]. However, our evaluations were focused on the less toxic compounds only (**1**–**5** and **7**), among which only aerophobin-1 (**1**) induced a significant increase in vertebral mineralization rate in zebrafish embryos/larvae when proposed at the non-toxic and relatively low concentration of 100 nM. Interestingly, the non-monotonic dose-response profile in osteogenesis experiments of aerophobin-1(**1**) was similar to that observed for the crude acetone *A. aerophoba* extract, supporting the hypothesis that **1** represents the main component in the extract responsible for the osteogenic effect. On the other hand, the anti-osteogenic effects detected at higher concentrations for both pure aerophobin-1 (**1**) and the crude extract from the sponge could be due to the exceeding of the toxicity threshold, with consequent inhibition of several developmental mechanisms in zebrafish larvae.

In conclusion, the use of zebrafish larvae as a mineralization model for the evaluation of bone development allowed for the fast identification of *A. aerophoba* as the source of a promising pro-osteogenic agent, aerophobin-1 (**1**). The study paves the way for further evaluations on the molecular mechanisms involved in the modulation of osteogenesis in embryos and on adult zebrafish specifically aimed at further supporting the use of **1** as an anti-osteoporotic drug. The discovery of new and more safe drugs that target bone depletion human diseases is a challenge for the scientific community but also an imperative issue for the health system. Indeed, the increasing number of patients with skeletal disorders, in part as a consequence of longer life expectancy, and to whom medical assistance has to be guaranteed [29,30], represents a severe economic burden for the whole society.

## 4. Materials and Methods

### 4.1. General Experimental Procedures

Optical rotations were measured on a Jasco P-2000 digital polarimeter. ESIMS were performed on a Micromass Q-TOF MicroTM coupled with an HPLC Waters Alliance 2695. The instrument was calibrated by using a PEG mixture from 200 to 1000 MW (resolution specification 5000 FWHM, deviation < 5 ppm RMS in the presence of a known lock mass). High-resolution mass spectra (HRESIMS) were acquired on a Q-Exactive hybrid quadrupole-orbitrap mass spectrometer (Thermo Scientific, San Jose, CA, USA). NMR experiments were recorded at the ICB-NMR Service Centre. Chemical shifts values are reported in ppm and referenced to internal signals of residual protons (CD_3_OD, ^1^H δ 3.34, ^13^C 49.0 ppm). 1D and 2D NMR spectra were acquired on a Bruker Avance-400 spectrometer using an inverse probe fitted with a gradient along the *Z*-axis, on a Bruker Avance III HD 400 MHz spectrometer equipped with a CryoProbe Prodigy, and on a DRX 600 spectrometer (600 MHz for 1H, 150 MHz for 13C) equipped with a three-channel inverse (TCI) CryoProbe. Sephadex LH-20 (GE Healthcare Bio-Sciences AB, Uppsala, Sweden) chromatography was performed on an open glass column (150 cm length, 3.0 cm diameter). Silicagel chromatography was performed using pre-coated Merck F254 plates (TLC) and Merck Kieselgel 60 powder (70–230 mesh). The spots on TLC were visualized under UV light (254 nm) and then sprayed with 10% H_2_SO_4_ in water, followed by heating.

### 4.2. Biological Material

The sponge *A. aerophoba* was collected by scuba diving at a depth of 12 m along the coast of Milos Island, Greece, during August 2014. Biological material was immediately frozen and then transferred to ICB in Naples, where it was kept at −2 °C until extraction. The sponge was identified by E. Mollo. A voucher sample of *A. aerophoba* (code Milos-P1) is available for inspection at ICB.

### 4.3. Extraction and Isolation Procedures

A frozen sample of *A. aerophoba* (8 g, dry weight) was chopped into small pieces and immediately immersed in acetone (4 × 200 mL). Extractions were aided by using an ultrasound bath. The organic solvent from these extractions was combined, filtered, and evaporated under reduced pressure to provide 1.25 g of crude extract. The extract (1.2 g) was loaded into a Sephadex LH-20 column equilibrated with MeOH, collecting 20 fractions with 100% MeOH and the last 8 using a mixture of MeOH/H_2_O (1:1). The resulting 28 fractions were combined on the basis of their chromatographic homogeneity to afford twelve main fractions, from A to N, which were analyzed by ^1^H NMR. Aerophobin-1 (**1**) and aerophobin-2 (2) were present in a mixture with other components in both fractions C (60 mg) and D (102 mg). The latter was further subjected to a silicagel column chromatography by using an increasing polarity gradient of MeOH in CHCl_3,_ giving three subfractions (D5-D7) containing aerophobin-1 (**1**) and one (D9) containing aerophobin-2 (**2**). A portion (5.0 mg) of the main aerophobin-1 (**1**) containing fraction D6 (20.0 mg) was then purified on a semipreparative TLC plate using as mobile phase CHCl_3_/MeOH 8:2. The UV band at R*f* 0.3 scratched from the plate contained pure aerophobin-1 (**1**, 4.0 mg). Similarly, fraction D9 (21 mg) was as well purified on a semipreparative TLC plate using a mobile phase a mixture of CHCl_3_/MeOH 7:3 and UV absorbing spot at R*f* 0.3, affording pure aerophobin-2 (**2**, 3.5 mg). ^1^H NMR analysis of fraction E (78.2 mg) revealed the presence of (+)-aeroplysinin-1 (**3**), which was obtained as a pure compound (9.0 mg) by semipreparative TLC chromatography (R*f* 0.75, CHCl_3_/MeOH 8:2). Fraction F (55.0 mg) was found to contain compound **4** by 1H NMR analysis. The subsequent purification of this fraction by semipreparative TLC (R*f* 0.65 in CHCl_3_/MeOH 8:2) yielded 8.5 mg of pure **4**. Fraction G (500.0 mg) resulted in being a complex mixture by NMR. A portion (100 mg) of this fraction was loaded onto a semipreparative SiO_2_ TLC plate in CHCl_3_/MeOH 9:1 to obtain pure hydroquinone derivative **5** (5.0 mg), fistularin-3 (**6**, 25.0 mg), and LLPAA216 (**7**, 8.0 mg). Finally, fraction H (58 mg) was subjected to TLC chromatography on a plate to yield uranidine (**8**, 20.1 mg).

### 4.4. Ethics Statement

In vivo experiments have been performed in the Zebrafish Laboratory (IRCCS R. Galeazzi, GSD Foundation, Milan, Italy) according to Italian and European guidelines on research (EU Directive 2010/63/EU). Tests on zebrafish and all protocols used in this study were authorized by the Ministry of Health (Italy) with n. 805/2021-PR.

### 4.5. Animals

*Danio rerio* of AB strain was housed in ZEBTEC© Bench Top System (Tecniplast) and maintained at 28 °C under standard conditions [31]. Embryos have been obtained by single pair of adult fish and, before experimentation, have been checked for general health conditions under a light stereomicroscope as described [32].

### 4.6. Zebrafish Treatment

Embryos were maintained at 28 °C in a dark incubator in standard growing medium (E3 medium, 5 mM NaCl, 0.17 mM KCl, 0.33 mM CaCl_2_, 0.33 mM MgSO_4_) from 1 hpf up to 5 dpf. Crude extract as well as pure compounds were dissolved first in DMSO (99.9% Sigma) and then diluted in E3 medium to obtain serial concentrations as follows. Both the crude extract and pure compounds were diluted 10 times starting from 0 to 300 mg/L and from 0 to 100 µM, respectively. Each concentration was assayed on 10 embryos in triplicate. Control (CTR) embryos were exposed to an amount of DMSO equivalent to the maximum concentration used to test the compounds. We preliminarily tested also E3 medium alone that does not show any alteration both in general embryo development and mineralization (data not shown). During the experiments, embryos were observed daily under a light/fluorescence stereomicroscope (SZX-ZB7 Olympus) to follow their general health conditions and vitality and to set up times and concentrations of crude extract and pure compounds that provide teratogenic and/or toxic effects on them.

### 4.7. Histochemical Analysis

Embryos were euthanized using a 300 mg/L tricaine methanesulfonate (Merk Life Science S.r.l., Milano, Italy) solution [33] and fixed in 3.5% formaldehyde/0.1 M sodium phosphate buffer. Soon after, double acid-free staining with Alcian Blue 8GX (Sigma) and Alizarin red S (ARS, Sigma) [34] was applied to stain cartilage and bone tissue, respectively. The quantification of osteogenesis level was measured as vertebral mineralization rate (N.V./L.), calculated as the number of mineralized vertebral bodies (N.V., positive for alizarin red S staining) normalized for the length of each embryo (L.).

All embryos were examined under a light/fluorescence stereomicroscope (SZX-ZB7 Olympus), acquiring images with a Discovery CH30 camera (Tiesselab), which were analyzed with ISC Capture software to take measurements.

### 4.8. Statistical Analysis

Data of each experiment carried out on 10 embryos in triplicate were used to calculate the mean value expressed as mean of the means of the 3 independent experiments ± standard deviation versus control. Data were plotted on SigmaStat 3.5 software (San Jose, CA, USA) and subjected to Student’s *t*-test with all significance values set at *p* < 0.05 (*), *p* < 0.01 (**), and *p* < 0.001 (***).

## Figures and Tables

**Table 1:** Toxic and osteogenic effects on developing embryos/larvae (PE, pericardial edema; NE, not evaluated).

Sample	Significant Toxic Effects		Pro-Osteogenic Activity Minimum Dose Effect
	Concentration	Timing	
Acetone extract	≥3 mg/L	5 dpf	3 µM
**1**	≥1 μM	5 dpf	100 nM
**2**	none (until 100 µM)	5 dpf	none
**3**	≥10 pM	5 dpf	none
**4**	≥1 pM	5 dpf	none
**5**	≥10 pM	5 dpf	none
**6**	≥10 pM	48 hpf (PE)	NE
**7**	none (until 100 µM)	5 dpf	none
**8**	≥1 pM	1 hpf	NE

## Data Availability

Data are contained within the article or supplementary files.

## Supplementary Materials

The following supporting information can be downloaded at: https://www.mdpi.com/article/10.3390/md20020135/s1, Figure S1: 1H NMR spectrum of aerophobin- 1 (1) (400 MHz, MeOD); Figure S2: ESI-MS spectrum of aerophobin- 1 (1); Figure S3: 1H NMR spectrum of aerophobin- 2 (2) (400 MHz, MeOD); Figure S4: ESI-MS spectrum of aerophobin- 2 (2); Figure S5: 1H NMR spectrum of (+)-aeroplysinin-1 (3) (400 MHz, CDCl3); Figure S6: ESI-MS spectrum of (+)-aeroplysinin-1 (3); Figure S7: 1H NMR spectrum of compound 4 (400 MHz, MeOD); Figure S8: ESI-MS spectrum of compound 4; Figure S9: 1H NMR spectrum of compound 5 (400 MHz, MeOD); Figure S10: ESI-MS spectrum of compound 5; Figure S11: 1H NMR spectrum of fistularin- 3 (6) (600 MHz, MeOD); Figure S12: ESI-MS spectrum of fistularin- 3 (6); Figure S13: 1H NMR spectrum of LL-PAA216 (7) (400 MHz, MeOD); Figure S14: ESI-MS spectrum of LL-PAA216 (7); Figure S15: 1H NMR spectrum of uranidine (8) (400 MHz, MeOD); Figure S16: ESI-MS spectrum of uranidine (8).
