# Supplementary material for: Aerophobin-1 from the Marine Sponge Aplysina aerophoba Modulates Osteogenesis in Zebrafish Larvae
Source: Mar Drugs. 2022 Feb 11;20(2):135. doi: 10.3390/md20020135 (PMC8880152; doi:10.3390/md20020135)
Supplement: Supplementary file 1 [file marinedrugs-20-00135-s001.zip › marinedrugs-1584702-supplementary.pdf]

Supplementary Materials

Aerophobin-1 from the marine sponge *Aplysina aerophoba* modulates osteogenesis in zebrafish larvae

Marta Carnovali [1], Maria Letizia Ciavatta [2], Ernesto Mollo [2], Vassilios Roussis[3], Giuseppe Banfi [1,4], Marianna Carbone[2,*], Massimo Mariotti [1,5*]

* Correspondence massimo.mariotti@unimi.it; Tel.: +39-0296474369 (M.M.); mcarbone@icb.cnr.it; Tel.: +39-0818675227 (M.C.)

Table of content:

S1. ^{1}H NMR spectrum of aerophobin- 1 (**1**) (400 MHz, MeOD)
S2. ESI-MS spectrum of aerophobin- 1 (**1**)
S3. ^{1}H NMR spectrum of aerophobin- 2 (**2**) (400 MHz, MeOD)
S4. ESI-MS spectrum of aerophobin- 2 (**2**)
S5. ^{1}H NMR spectrum of (+)-aeroplysinin-1 (**3**) (400 MHz, $CDCl_3$)
S6. ESI-MS spectrum of (+)-aeroplysinin-1 (**3**)
S7. ^{1}H NMR spectrum of compound **4** (400 MHz, MeOD)
S8. ESI-MS spectrum of compound **4**
S9. ^{1}H NMR spectrum of compound **5** (400 MHz, MeOD)
S10. ESI-MS spectrum of compound **5**
S11. ^{1}H NMR spectrum of fistularin- 3 (**6**) (600 MHz, MeOD)
S12. ESI-MS spectrum of fistularin- 3 (**6**)
S13. ^{1}H NMR spectrum of LL-PAA216 (**7**) (400 MHz, MeOD)
S14. ESI-MS spectrum of LL-PAA216 (**7**)
S15. ^{1}H NMR spectrum of uranidine (**8**) (400 MHz, MeOD)
S16. ESI-MS spectrum of uranidine (**8**)

S1

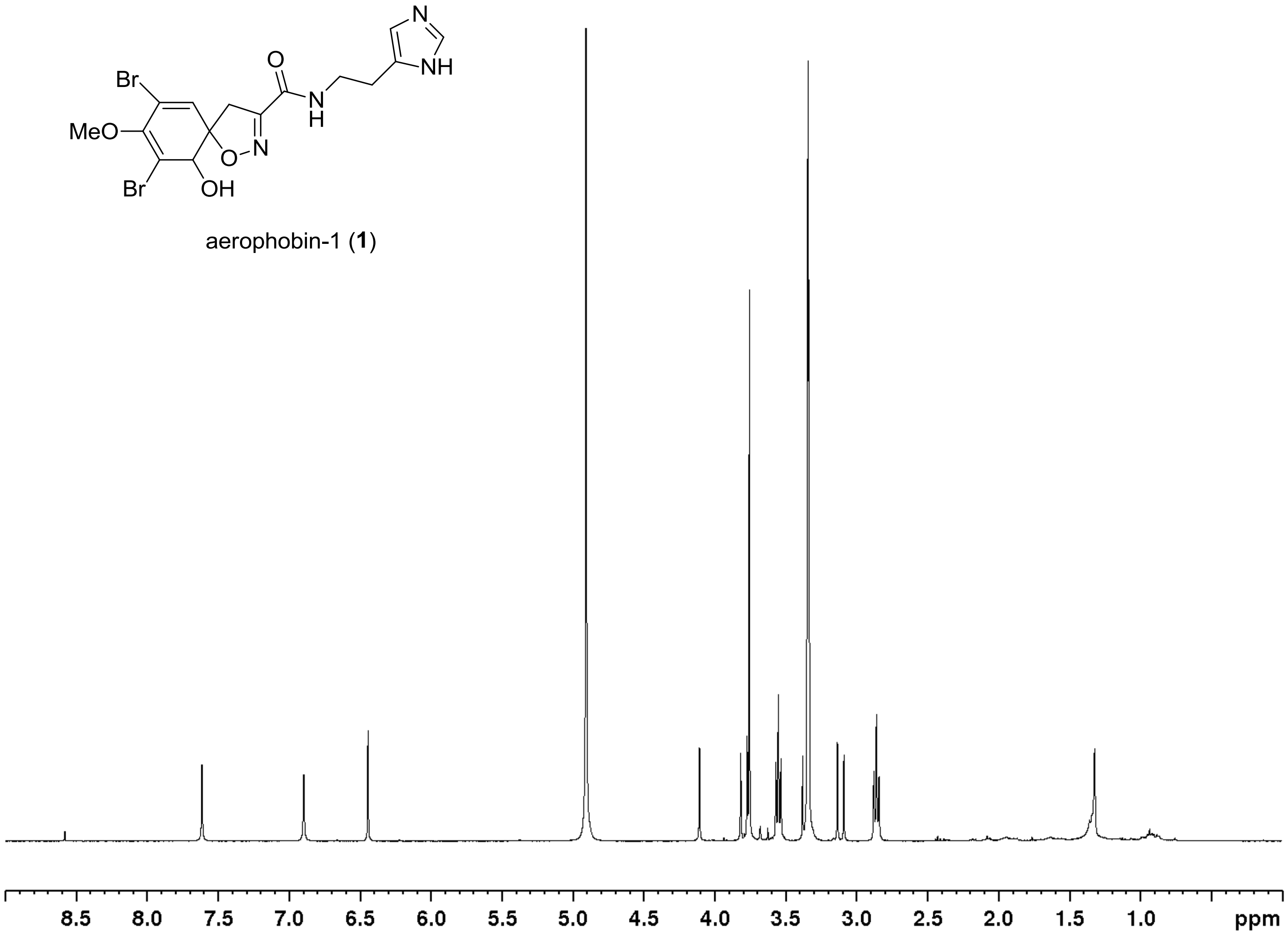

^{1}H NMR spectrum of aerophobin- 1 (**1**) (400 MHz, MeOD)

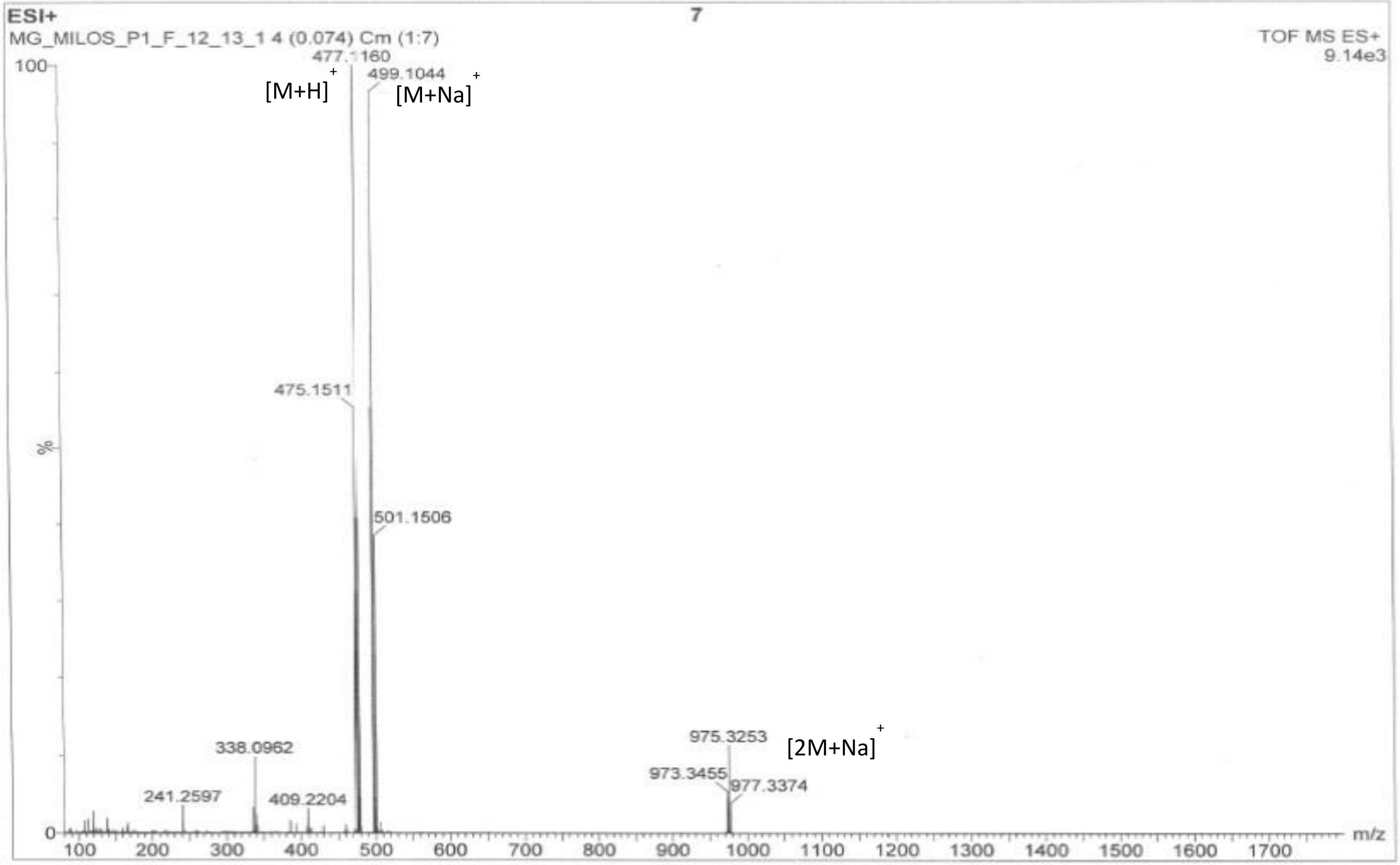

ESI-MS spectrum of aerophobin- 1 (**1**)

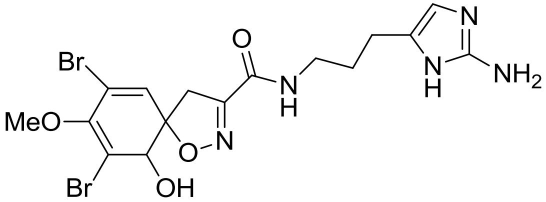

aerophobin-2 (**2**)

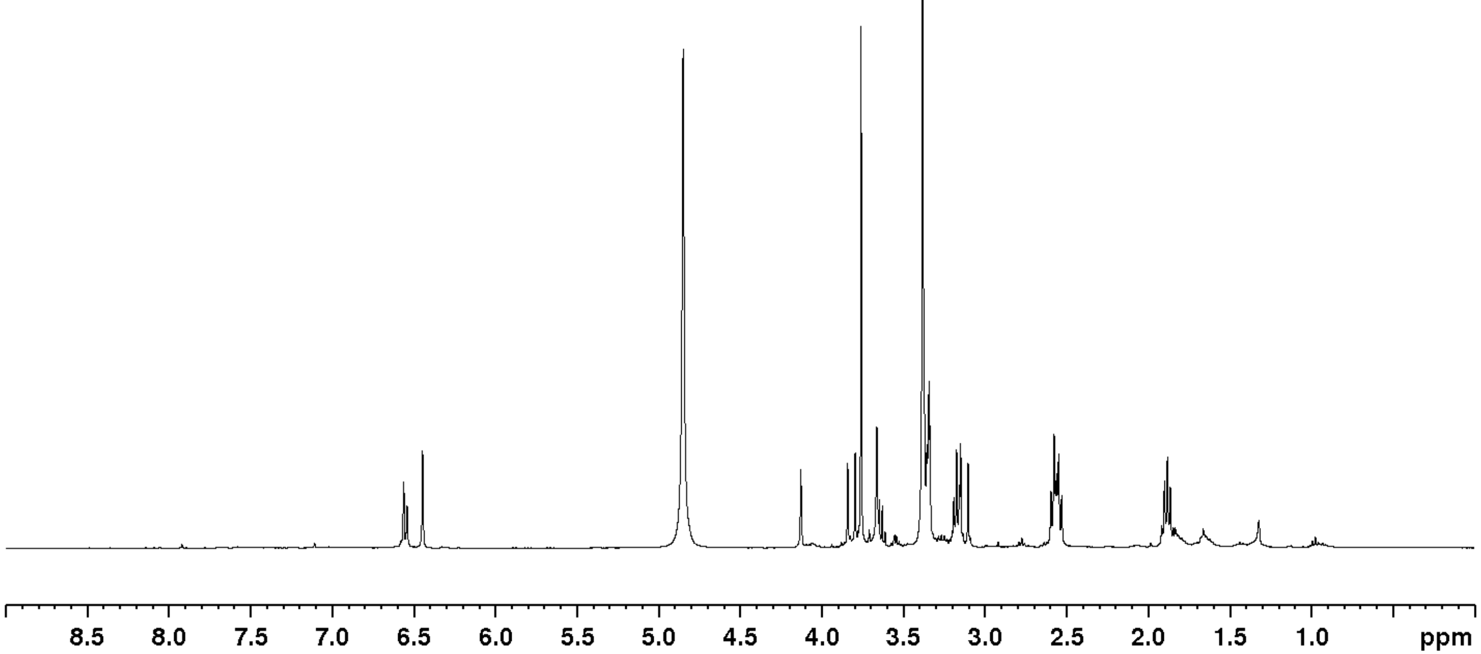

^{1}H NMR spectrum of aerophobin- 2 (**2**) (400 MHz, MeOD)

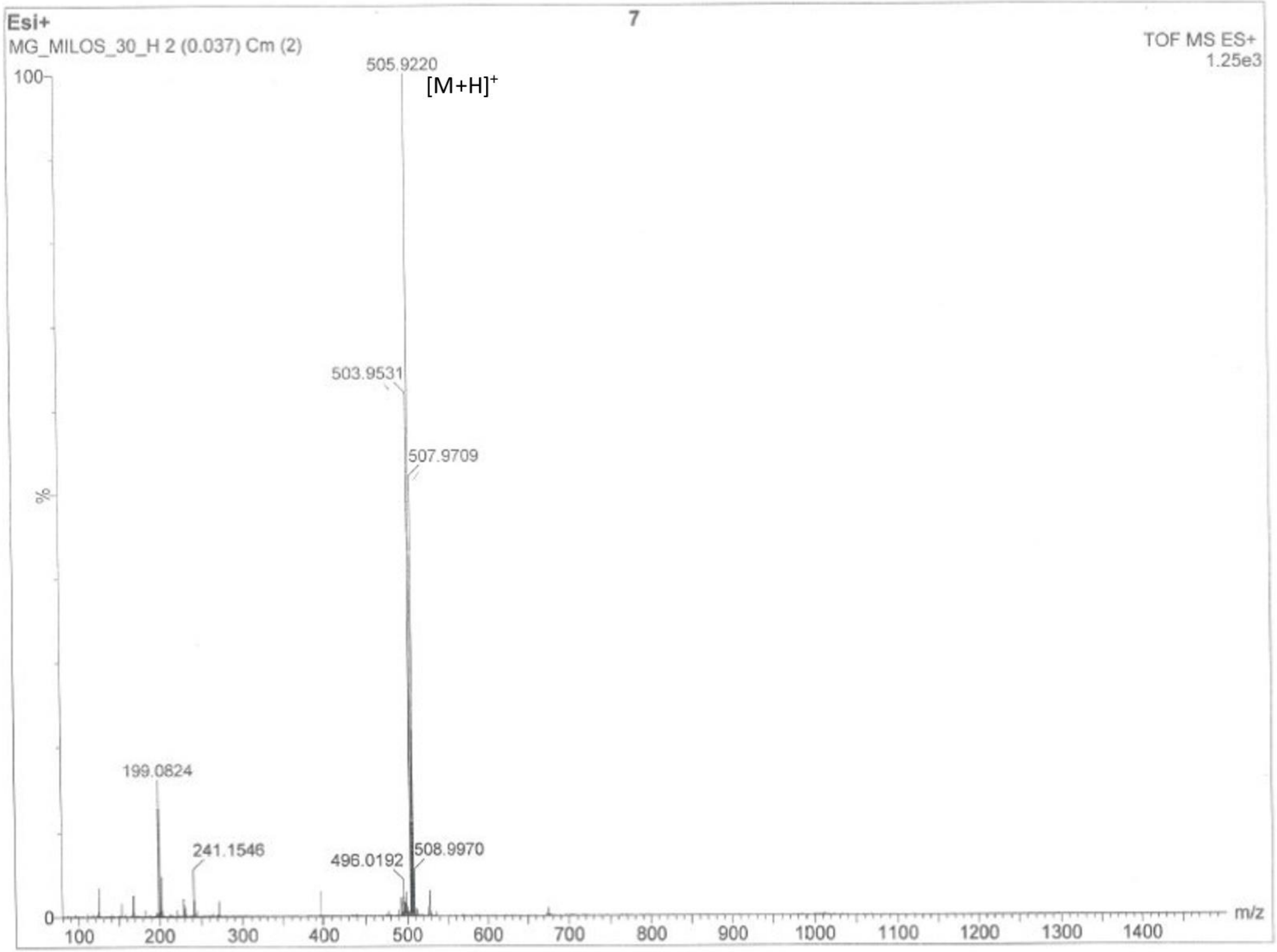

ESI-MS spectrum of aerophobin- 2 (**2**)

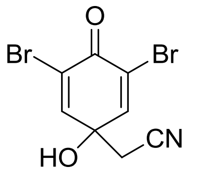

aeroplysinin-1 (**3**)

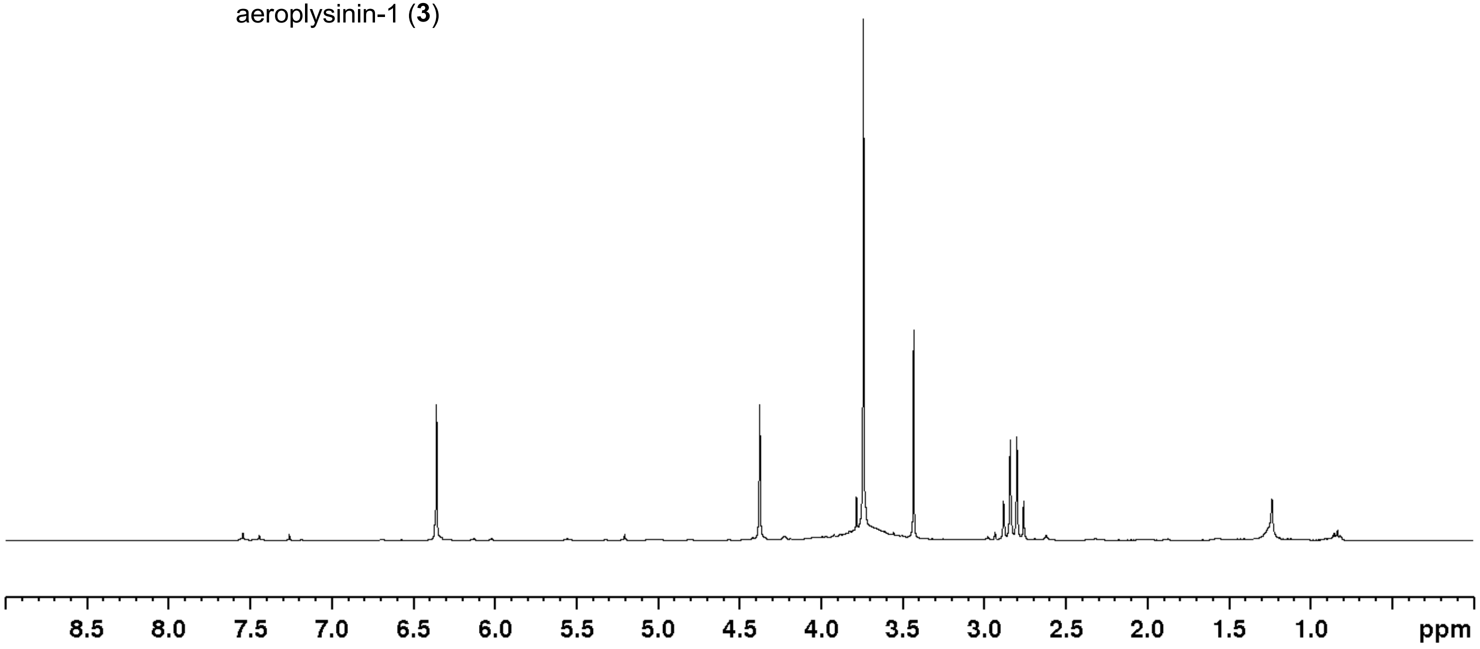

^{1}H NMR spectrum of (+)-aeroplysinin-1 (**3**) (400 MHz, $CDCl_3$)

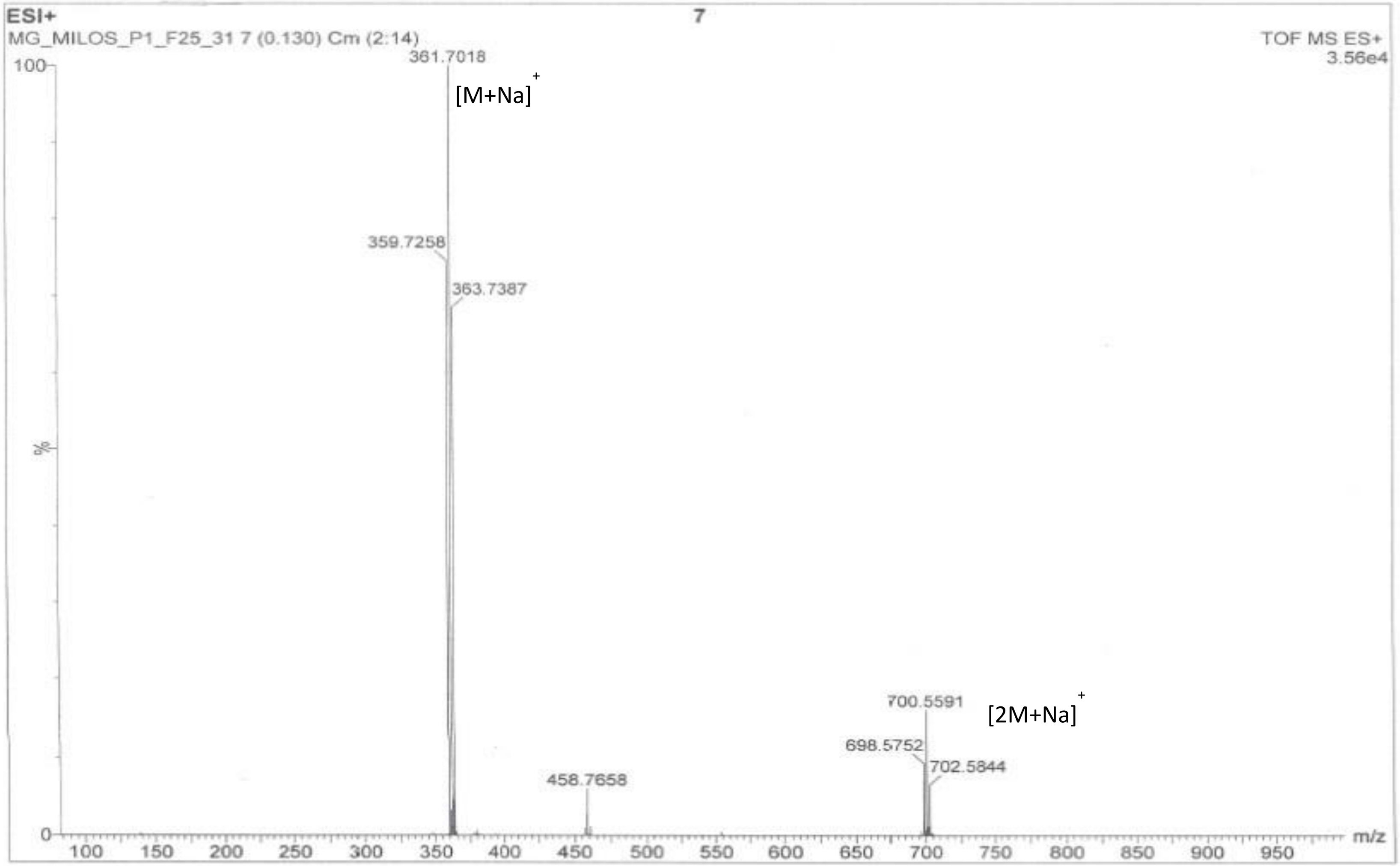

ESI-MS spectrum of (+)-aeroplysinin-1 (**3**)

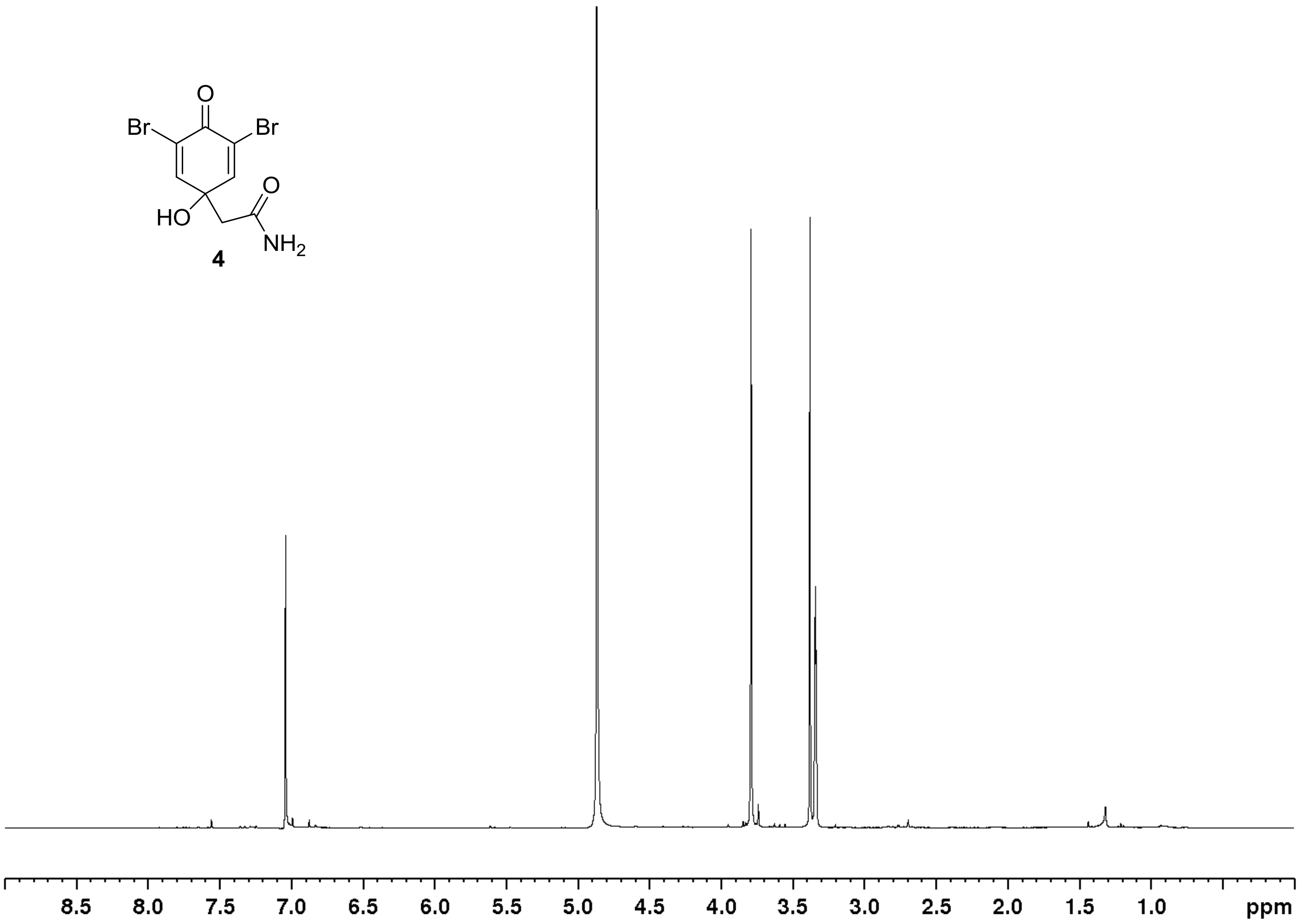

^{1}H NMR spectrum of compound **4** (400 MHz, MeOD)

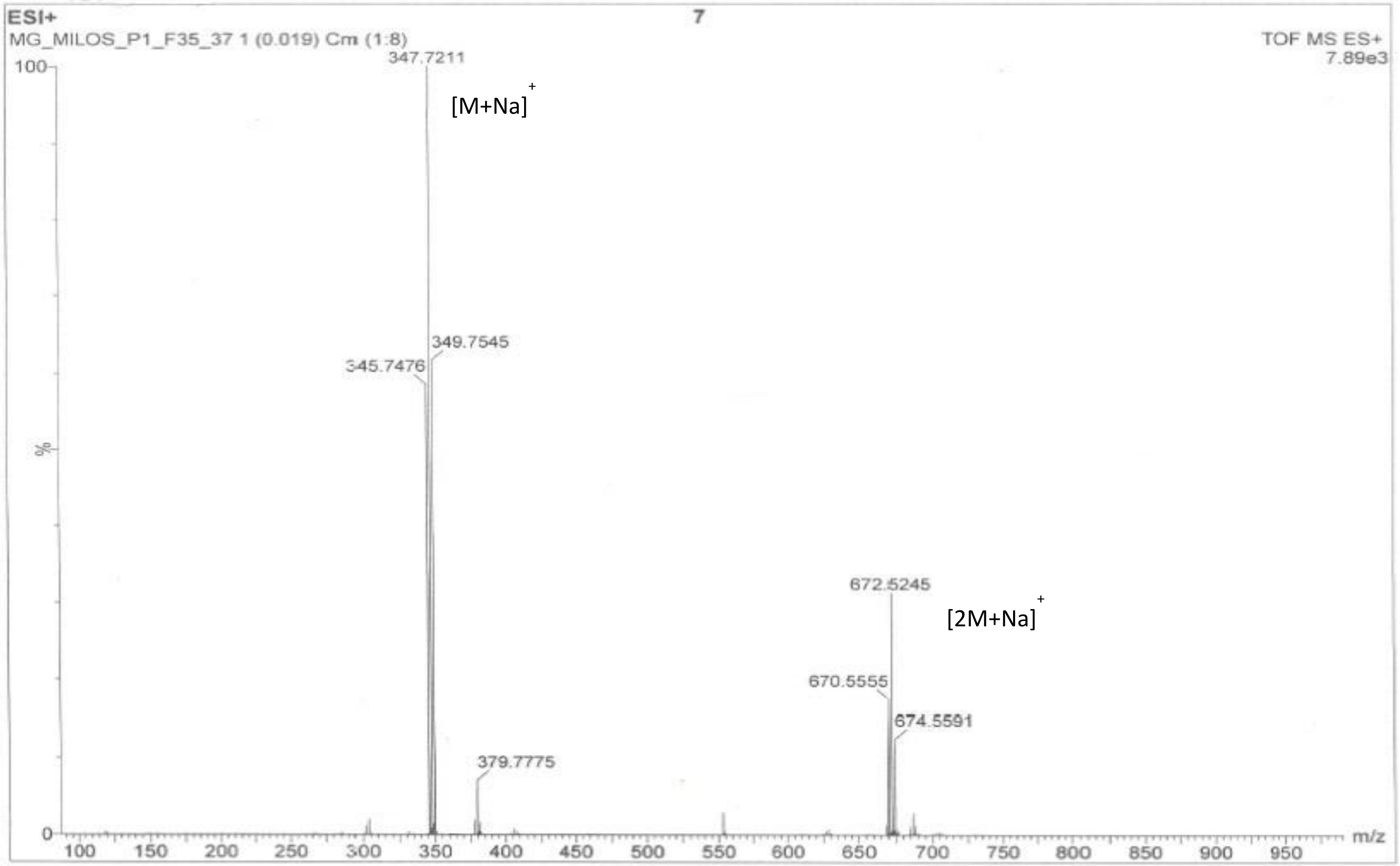

ESI-MS spectrum of compound **4**

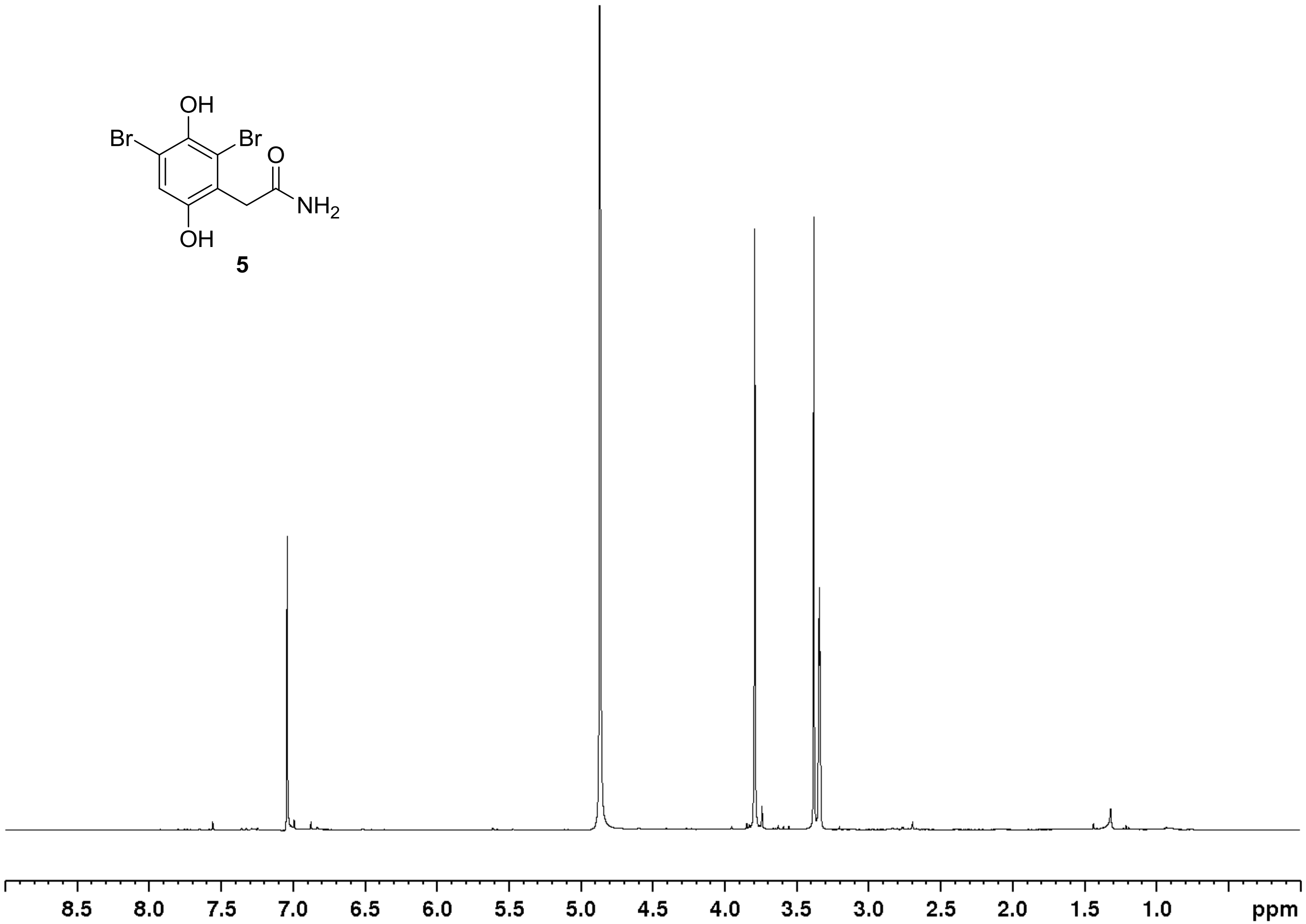

^{1}H NMR spectrum of compound **5** (400 MHz, MeOD)

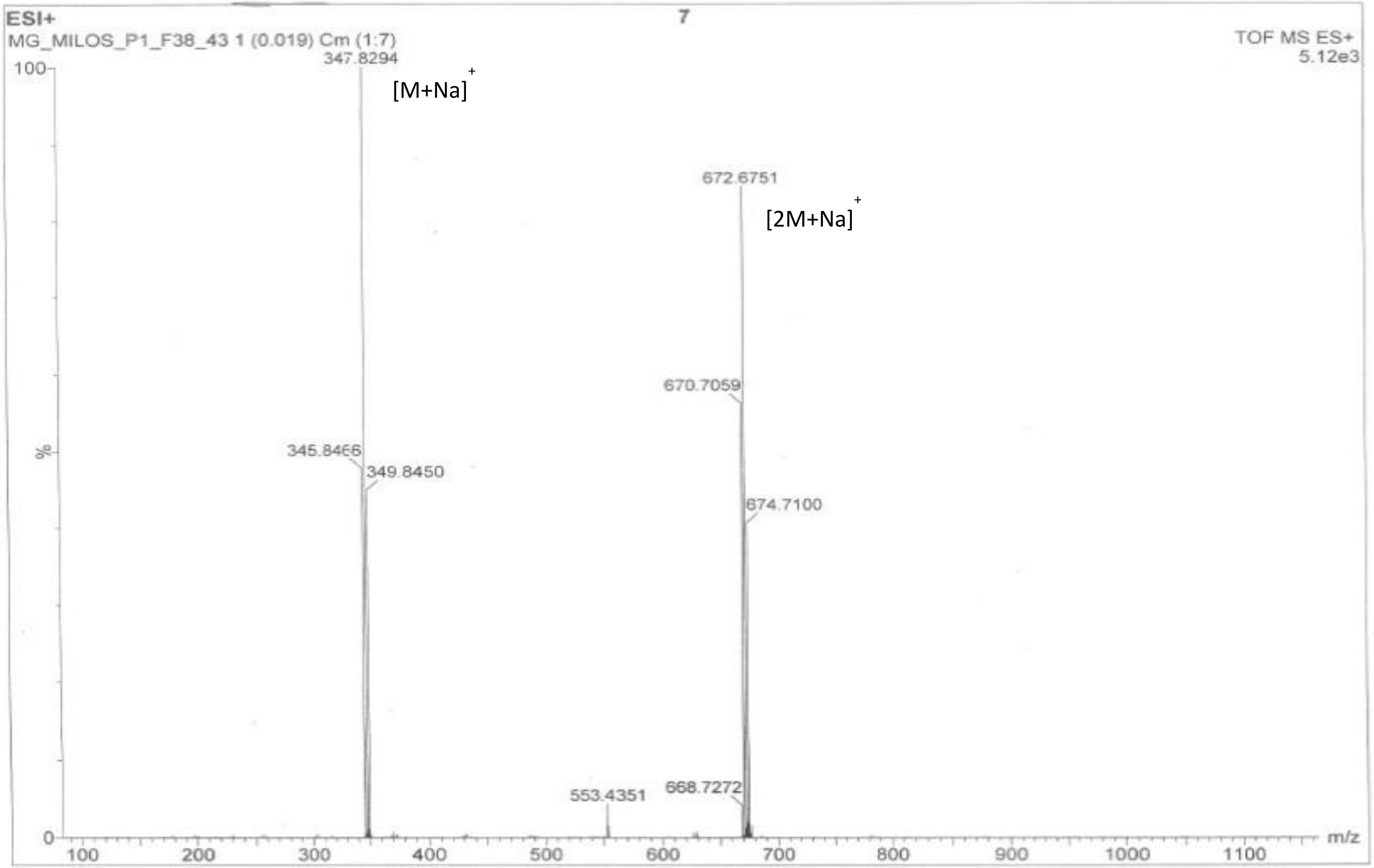

ESI-MS spectrum of compound **5**

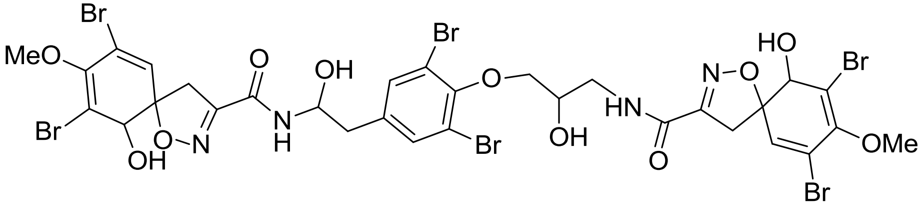

fistularin-3 (**6**)

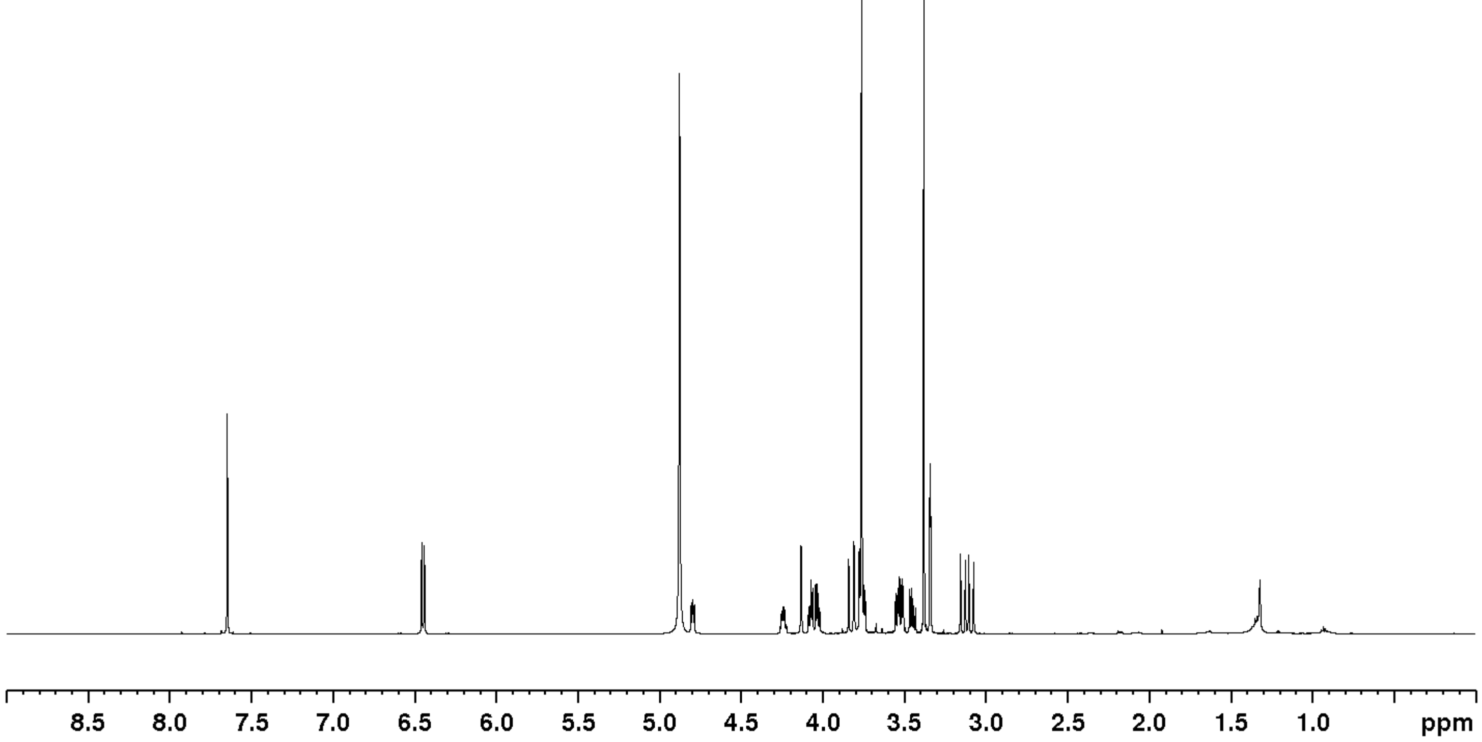

^{1}H NMR spectrum of fistularin- 3 (**6**) (600 MHz, MeOD)

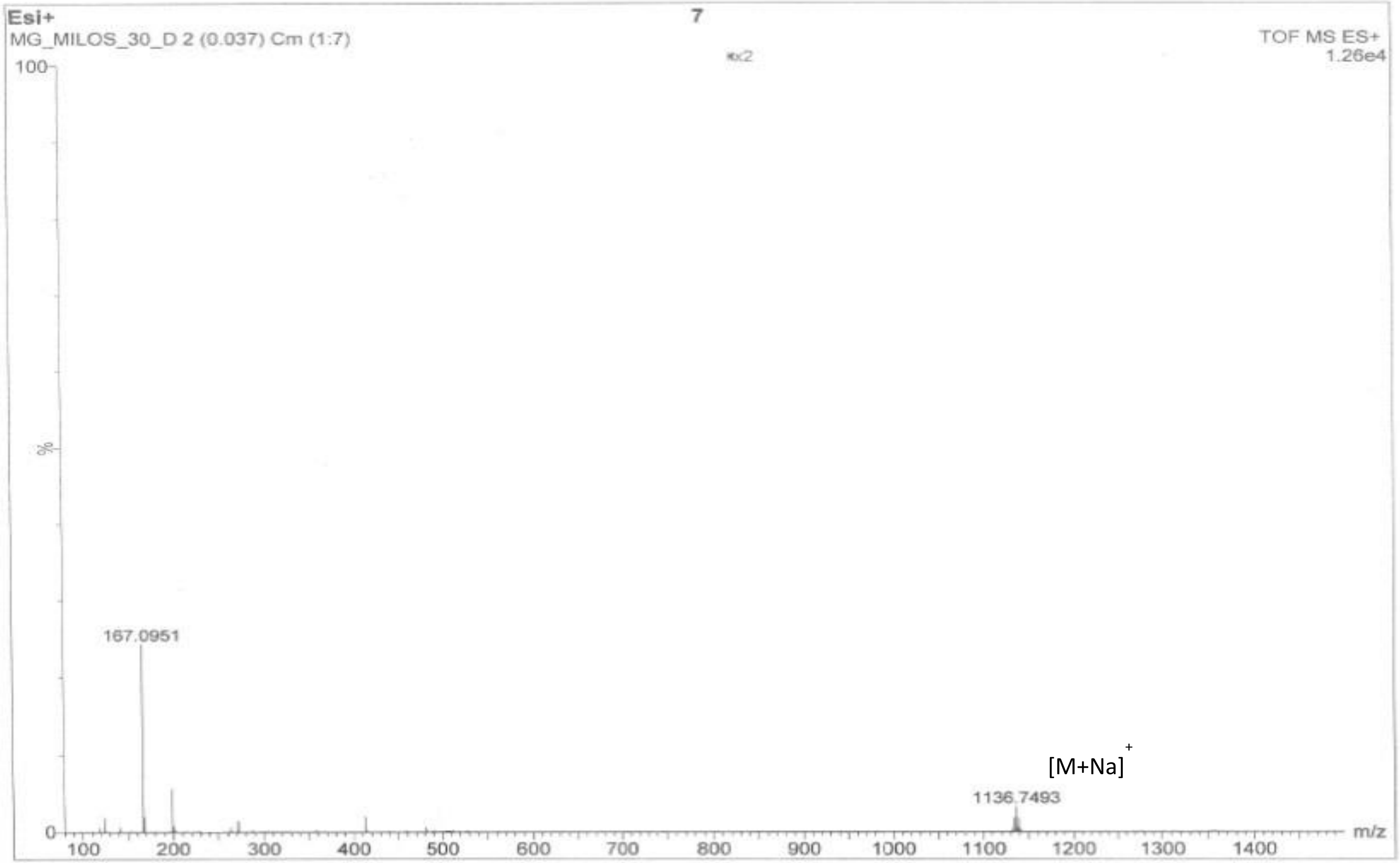

ESI-MS spectrum of fistularin- 3 (**6**)

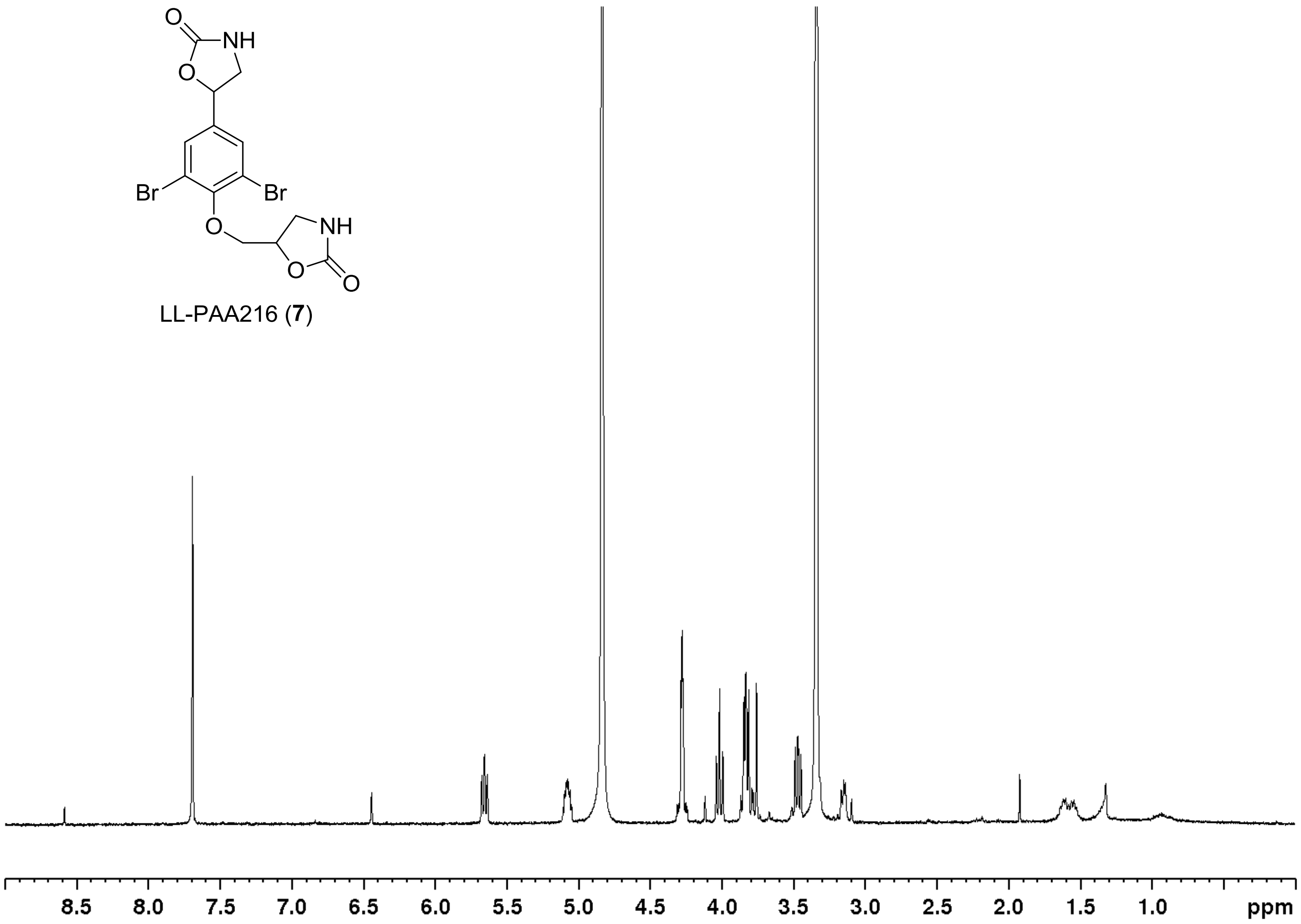

^{1}H NMR spectrum of LL-PAA216 (**7**) (400 MHz, MeOD)

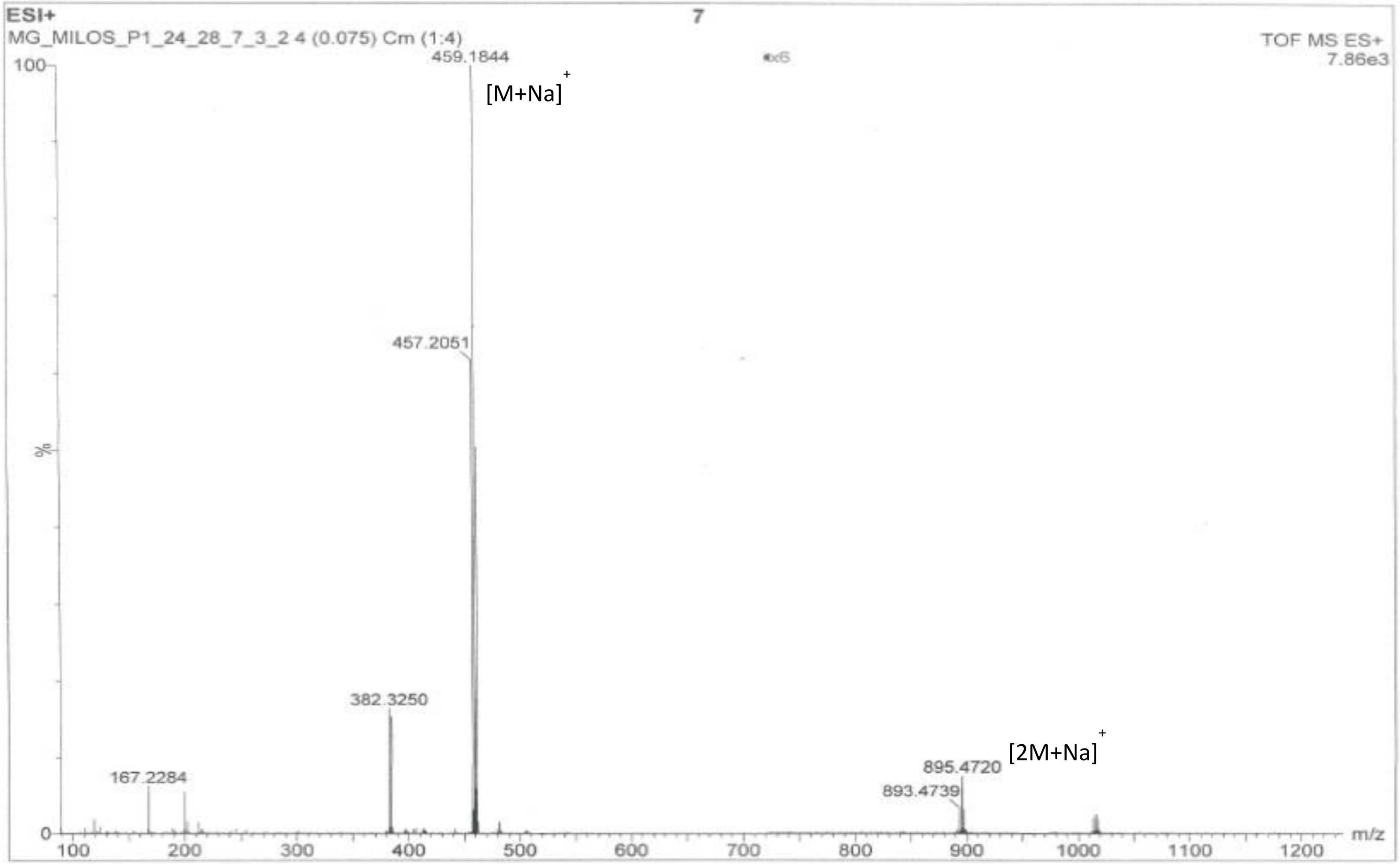

ESI-MS spectrum of LL-PAA216 (**7**)

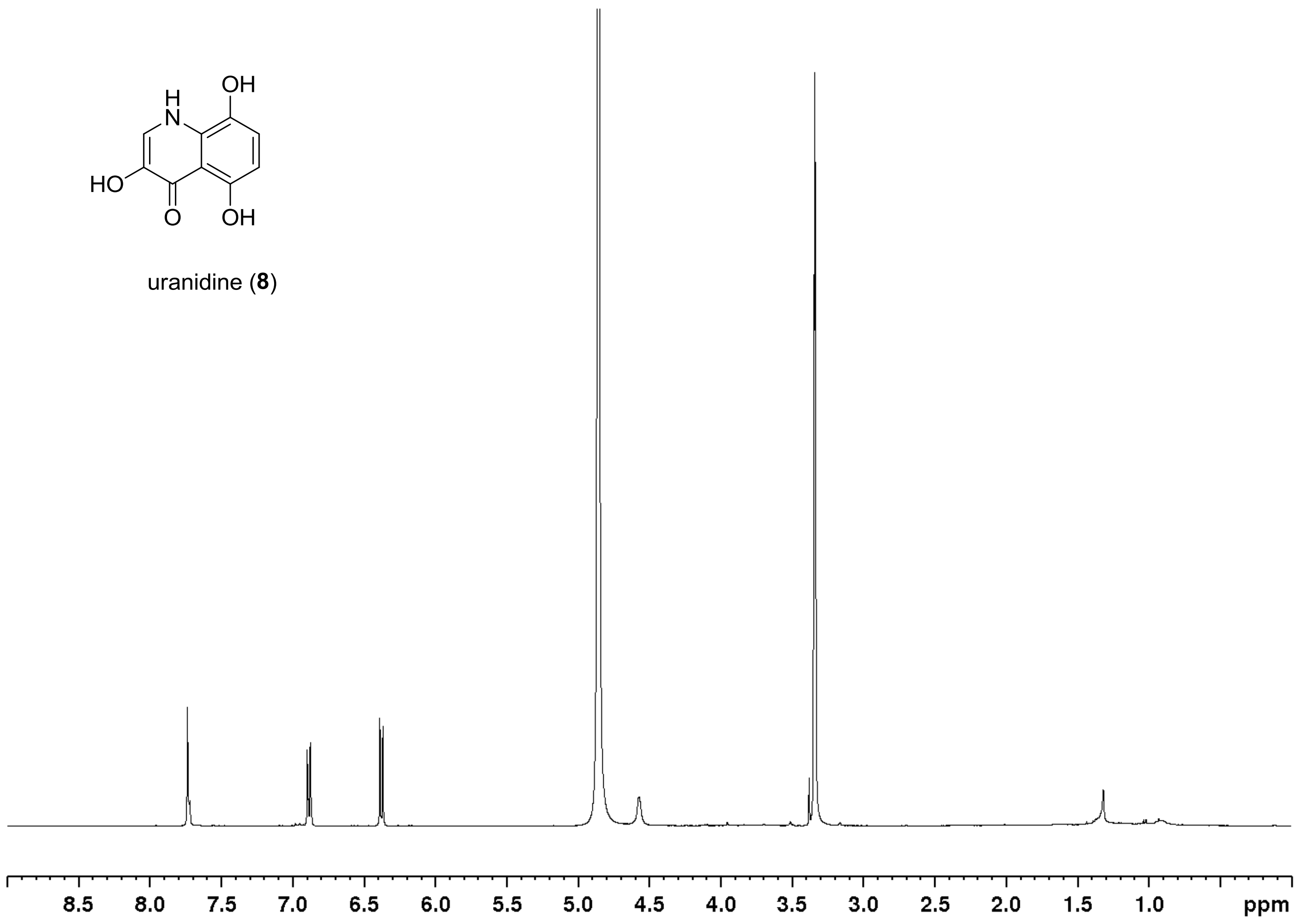

^{1}H NMR spectrum of uranidine (**8**) (400 MHz, MeOD)

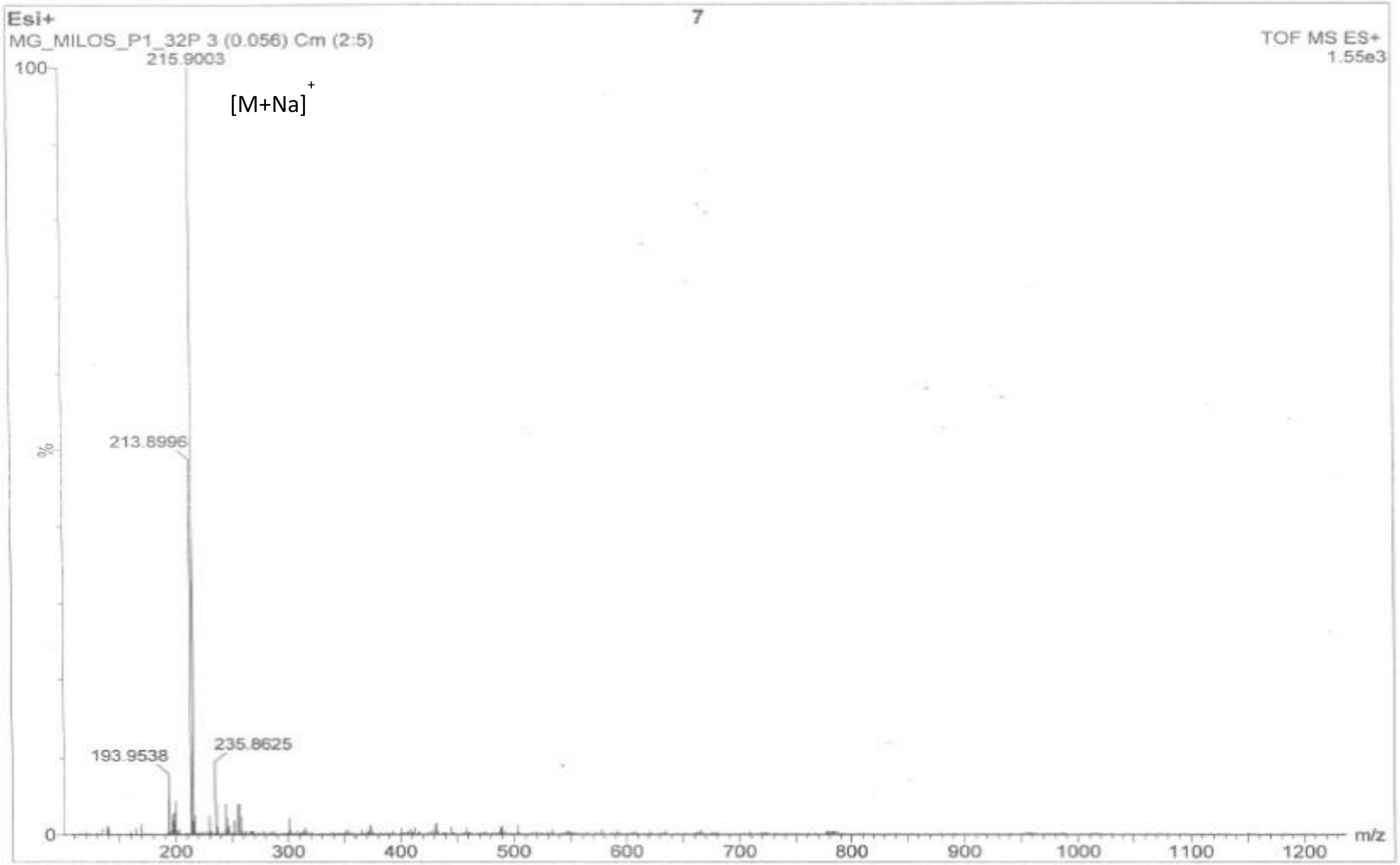

ESI-MS spectrum of uranidine (**8**)
